# Electrochemical nasal nitric oxide measurement during laryngeal mask ventilation as primary ciliary dyskinesia screening

**DOI:** 10.1183/23120541.01018-2024

**Published:** 2025-11-10

**Authors:** Tobias Lipek, Maike vom Hove, Mandy Vogel, Konrad Platzer, Freerk Prenzel

**Affiliations:** 1 Department of Pediatrics, University of Leipzig Medical Center, Leipzig, Germany; 2 University of Leipzig Medical Center, Leipzig Interdisciplinary Center for Allergy (LICA), Leipzig, Germany; 3 University of Leipzig Medical Center, Center for Pediatric Research Leipzig, LIFE Child, Leipzig, Germany; 4 Institute of Human Genetics, University of Leipzig Medical Center, Leipzig, Germany

## Abstract

**Introduction:**

Diagnosis of primary ciliary dyskinesia (PCD) can be challenging, especially in children of preschool age. Measurement of nasal nitric oxide (nNO) production helps in making the diagnosis. While chemiluminescence-based nNO measurement is sufficiently established, measuring nNO using electrochemical devices is common in older patients, although data on their repeatability and accuracy are scarce.

**Methods:**

This is a retrospective analysis of nNO measurements using an electrochemical technique (ECnNO) in 52 children undergoing bronchoscopy including 10 PCD cases. Using a novel approach to obtain electrochemical measurements in even very young children (n=31) (59.6%) were <5 years of age), ECnNO was measured while patients were in breath-hold during ventilation with laryngeal mask (ECnNO LAMA). For agreement, the intraclass correlation coefficient (ICC) was calculated. Precision is described by the measurements’ coefficient of variation.

**Results:**

ECnNO LAMA measurements yielded an overall substantial ICC of 0.974 (95% CI 0.972–0.990) with no statistical difference for patients over or under 5 years. The overall coefficient of variation was 0.116 and thus comparable to chemiluminescence-based measurements in this age group.

**Conclusion:**

The novel ECnNO LAMA technique is feasible and showed promising repeatability and precision in screening for PCD in children <5 years of age. While further studies are needed, this method may help diagnose PCD in young patients and simplify a troublesome differential diagnostic process.

## Shareable abstract

**Novel electrochemical NO measurement during laryngeal mask ventilation showed promising repeatability and precision in screening for PCD in children <5 years. This may help diagnose PCD in young patients and simplify differential diagnostics.** https://bit.ly/43kifHf

## Introduction

Primary ciliary dyskinesia (PCD) is an important differential diagnosis when reviewing young patients with chronic cough, hearing loss, chronic nasal congestion, unexplained neonatal respiratory distress, organ laterality defect, cardiac defects or bronchiectasis [1].

When suspecting PCD, clinicians face diagnostic challenges. Often, a variety of diagnostic procedures are needed to confirm or exclude PCD, including measurement of nasal nitric oxide (nNO), high-speed video microscopy analysis (HSVA), transmission electron microscopy (TEM), cell culture and genetic testing. Given its overall good negative predictive value, the European Respiratory Society (ERS) and the American Thoracic Society (ATS) both recommend nNO measurement as the first step in PCD diagnosis [2, 3]. As a screening tool, this measurement may reduce the burden of further invasive and costly investigations. Nonetheless, attention must be paid to technical detail; two different methods for nNO measurement are available.

Although the chemiluminescence technique has been extensively studied and used at larger tertiary centres, electrochemical nNO (ECnNO) testing is more widely used [4]. This is most likely the case because nNO measurements using chemiluminescence are many times more expensive than electrochemical measurements [5, 6], approximately EUR 50 000 *versus* EUR 3000 per device (values from own market research, for orientation only).

The main technical disadvantage of electrochemical measurement is the duration of constant airflow needed for valid measurements, making it practically impossible to use for children <5 years of age. Measurement of ECnNO requires ≥15 s, preferably 30 s, of sampling time [7, 8]. Using chemiluminescence techniques, it is possible to obtain valid measurements from an online display of nNO flow in only 3 s [5, 9], and the measurement may be completed even faster [10]. Furthermore, chemiluminescence measurements are possible during normal tidal breathing, during plateaus in breath-hold and during expiration against resistance. Measurements using electrochemical devices are more difficult to perform [11, 12]. Therefore, centres without access to chemiluminescence-based nNO (CInNO) devices face technical challenges in obtaining valid measurements, especially in young children.

In the device manual (NIOX VERO; Circassia AB, Uppsala, Sweden), only data from a company-funded study are available, and these were only published as a poster presentation [8, 13], while the registered study did not publish any results [14]. To our knowledge, no complete datasets on the accuracy, repeatability and precision of ECnNO measurements in children <5 years of age have been published. It is therefore not surprising that electrochemical measurement is not approved in the USA and clinicians question this method. This knowledge gap is addressed in the ERS’ recent technical standard on nNO measurement, in which the authors call for quality evidence regarding electrochemical analysers and sampling methods used in young children [4].

In this setting, we have introduced a new technique for measuring nNO with our electrochemical device, which is performed during ventilation with a laryngeal mask. The first test results were promising and could provide a practicable way to measure nNO. Hence, this retrospective pilot study aims to evaluate the repeatability of ECnNO measurement during laryngeal mask ventilation (ECnNO LAMA) in children.

## Material and methods

### Study subjects and setting

This monocentric retrospective comparative study was approved by the Research Ethics Committee of the Medical Faculty of the University of Leipzig (registration number 318/22 ek). The inclusion criteria were: scheduled bronchoscopy at our centre between February 2018 and January 2023; PCD as a potential differential diagnosis and therefore the clinical need to obtain nNO measurements; and written informed consent from the children's legal guardians for bronchoscopy and measurement of nNO, at least two measurements of the nNO value and general anaesthesia in accordance with local clinical standards. The only exclusion criterion was the presence of clear signs of a respiratory infection.

### Diagnostic criteria of PCD cases and controls

The final PCD diagnosis was made based on the criteria from the international PCD registry (www.pcdregistry.eu/) [15] and the ERS/ATS guidelines [2, 3], with special emphasis on genetic diagnostics. HSVA was not available at our centre during the study, but it was performed on two patients at a national reference centre. Patients were regarded as PCD cases and probable cases, respectively, if 1) the clinical findings were consistent with PCD, and 2) at least one of the following four examination procedures revealed findings indicating pathogenicity: TEM, immunofluorescence microscopy, detection of biallelic mutation by genotyping or HSVA (table 1 and supplementary material). Patients with other respiratory diseases who did not meet the above diagnostic criteria for PCD were controls. Immunofluorescence microscopy, genetic testing and TEM were performed at external reference centres. For analyses requiring a dichotomous classification of diagnostic certainty (descriptive statistics and receiver operating characteristic (ROC) analysis), cases and probable cases were treated as one group *versus* controls. See figure S4 for a flow chart of the study population.

**TABLE 1:** Characteristics of primary ciliary dyskinesia cases and probable cases

Patient number	Sex	Age at date of bronchoscopy (years)	Clinical features	TEM	IF	Genetic variants, protein effect, ACMG classification	Classification^#^	nNO measurements, ppb		
								1	2	3
**9**	F	0.1	Perinatal persistent atelectasis of right UL; HSVA showed ciliary beat frequency 6–8 Hz, beat pattern hyperkinetic with reduced amplitude^¶^	Significant reduction of outer and inner dynein arms	Normal	*DNAH5* (compound heterozygous), c.10815delT, p.(Pro3606Hisfs*23), pathogenic; c.8897C>T, p.(Thr2966Met), VUS^#^	Case	68	28	29
**11**	M	7.5	Situs inversus totalis, chronic pansinusitis, chronic otitis media, *Pseudomonas aeruginosa* lung infections, bronchiectasis right LL	Not done	Not done	*DNAI1* (homozygous), c.1162T>C, p.(Cys388Arg), likely pathogenic	Case	9	17	7
**22**	F	0.1	Term newborn with respiratory distress syndrome, congenital pneumonia	Normal	DNAH5 within cilia absent	*LRRC6* (compound heterozygous), c.79_80delTC, p.(Ser27Valfs*13), pathogenic; c.574C>T, p.(Gln192*), pathogenic	Case	28	45	66
**32**	F	17.2	Chronic rhinosinusitis with nasal polyposis, bronchiectasis in both LL, chronic otitis media with eardrum perforation; HSVA showed elevated ciliary beat frequency of >7 Hz^¶,+^	Normal	Normal	*DNAH11* (heterozygous variant), c.6680G>T, p.(Gly2227Val), VUS^#^	Probable case	42	35	38
**37**	M	0.7	Situs inversus totalis	Combined dynein arm defect	Sample quality insufficient	*CFAP52* (homozygous), c.1304delG, p.(Gly435Alafs*7, pathogenic	Case	38	29	32
**39**	M	3.6	Chronic bronchitis, CDH, chronic otitis media	Not done	DNAH5, defect of outer dynein arm	*DYX1C1* (compound heterozygous), c.583delA, p.(Ile195*), pathogenic; c.1052dup, p.(Glu352Glyfs*12), pathogenic	Case	33	32	30
**40**	F	17.1	Chronic otitis media, chronic mastoiditis, bronchiectasis in right ML and LL, infection by *Pseudomonas aeruginosa*	Normal	Sample quality insufficient	*DNAH11* (compound heterozygous variants), c.5778G +1G>A, p?, pathogen+c.9765A>T, p.(Leu3255Phe), VUS^#^	Probable case	363	360	361
**42**	M	10.5	Chronic bronchitis, bronchiectasis in ML, LL bilaterally, chronic rhinosinusitis, CDH	Not done	Sample quality insufficient	*DNAJB13* (homozygous), deletion of entire exon 1, pathogenic	Case	156	98	127
**45**	F	1.9	Full-term neonate with neonatal respiratory distress, recurrent pulmonary infections	Not done	DNAH5 mislocalisation compatible with defect of ODA	*SPAG1* (homozygous), c.2014C>T, p.(Gln672*), pathogenic	Case	37	37	39
**52**	M	0.5	Full-term neonate with neonatal respiratory distress, situs inversus totalis, CCTGA	Not done	Normal	Two variants in *HYDIN*^#^, VUS	Probable case	5	21	19

A list of the genes that were tested for can be found in the supplementary material. For immunofluorescence (IF), antibodies binding with DNAH5, GAS8 and RSPH9 were used. TEM: transmission electron microscopy; ACMG: American College of Medical Genetics and Genomics; nNO: nasal nitric oxide; F: female; UL: upper lobe; HSVA: high-speed video analysis; VUS: variant of uncertain significance; M: male; LL: lower lobe; CDH: conductive hearing loss; ML: middle lobe; ODA: outer dynein arm; CCTGA: congenitally corrected transposition of the great arteries. ^#^: see supplementary material; ^¶^: additional HSVA was performed at a national reference centre; ^+^: low nNO was confirmed by chemiluminescence technology (20.7 nL·min^−1^).

### nNO measurements

The examinations were performed in one of four similar operating theatres with automatic air supply and air conditioning. nNO was measured *via* an electrochemical device (NIOX VERO; Circassia AB, Uppsala, Sweden) with a flow rate of 0.3 L·min^−1^. The ambient NO concentration was checked during all measurements and was always <5 ppb (below the detection limit of the device), which is well below the suggested threshold for absence [5]. With a controlled fresh air supply of 1600 m^3^·h^−1^ in the operating room, stable conditions for ambient air were assumed.

With adequate sedation, a laryngeal mask was inserted to exclude nasal exhalation during the nNO measurement. Standardised preparation for bronchoscopy was initiated, including continuous measurement of oxygen saturation and monitoring of exhaled carbon dioxide_._ The duration of each measurement was 30 s with breath-hold and paused ventilation (figure S1). Between measurements, patients were ventilated to ensure an oxygen saturation of >97% and normal expiratory carbon dioxide. Three measurements were attempted, but in the event of technical problems (*e.g.* sampling too short, blocked nose or internal device error), a third measurement was not repeated to avoid prolonged sedation.

### Data analysis

Descriptive statistics are given as mean±sd for continuous variables and counts (percentages) for categorical variables.

To assess within-patient variability, *i.e.* agreement of the repeated nNO measurements per patient, we determined the intraclass correlation coefficient (ICC), also known as the reliability index. The ICC is estimated by applying variance components [16]. The categories chosen for the ICC for the strength of agreement are based on the categorisation by McBride [17] of Lin's concordance correlation coefficient for continuous variables: >0.99 (almost perfect), 0.95–0.99 (substantial), 0.90–0.95 (moderate) and <0.90 (poor).

We calculated the precision of the measurement per subject using the coefficient of variation (CV). Group comparison of the CVs was carried out using an unpaired t-test. The nNO thresholds were determined using ROC analysis based on the mean value of the nNO measurements obtained per person.

The significance level was set to α=0.05. Statistical analyses were performed using R (www.R-project.org) for ROC, CV and ICC; jamovi (www.jamovi.org) was used for descriptive statistics. In R, the package pROC [18] was used for ROC analysis, the package cccrm [19] was used for calculation of the ICC and ggplot2 [20] was used for visualisation.

## Results

**FIGURE 1:**
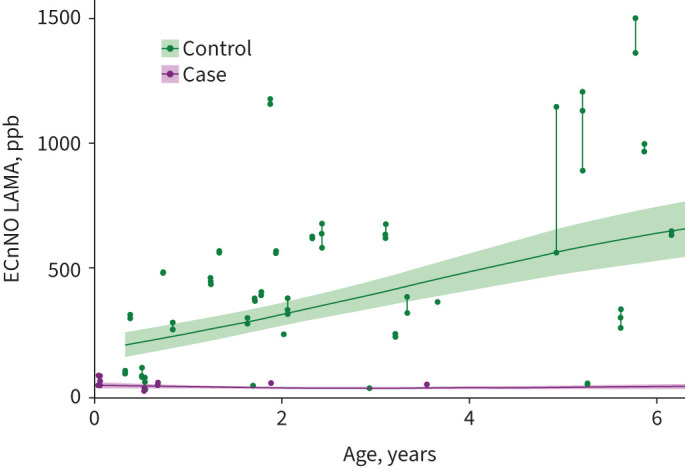
Electrochemical nasal nitric oxide measurement during laryngeal mask ventilation (ECnNO LAMA) *versus* age in cases and controls. Shaded areas represent confidence intervals. The *x*-axis is clipped at approximately 6* *years of age. See figure S2 for the whole cohort.

A total of 70 patients were examined, with 52 patients meeting the inclusion criteria. For 18 patients, the clinical records showed signs of upper respiratory infection (URI), leading to their exclusion. Of the children examined, 10 were diagnosed with confirmed or probable PCD and 42 were classified as a control group with other respiratory diseases. The characteristics of the study population are shown in table 2, and the details of the patients with PCD are listed in table 1. Age distribution *versus* PCD status is also shown in figure 1. Three nNO measurements were obtained in a total of 29 (55.8%) patients, and two measurements were recorded in 23 patients.

**TABLE 2:** Study population characteristics (n=52)

**Age at measurement**	
Overall	4.85±4.68 (range 0.05–17.6)
<5 years	31 (59.6%)
≥5 years	21 (40.4%)
Age in PCD group, years	5.91±6.86
Age in control group, years	4.60±4.07
**Sex**	
Female	23 (44.2%)
Male	29 (55.8%)
**Final diagnosis of PCD**	
Yes	10 (19.2%)
No	42 (80.8%)

Data are presented as mean±sd and n (%) of patients. PCD: primary ciliary dyskinesia.

### Repeatability of ECnNO LAMA measurements

**FIGURE 2:**
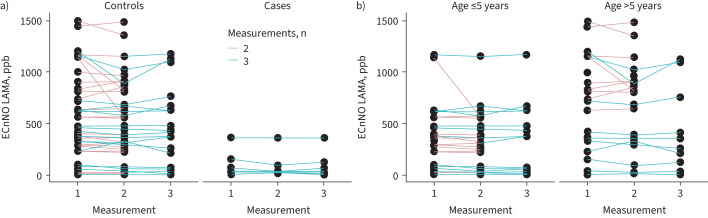
Plots showing all measurements in all subjects studied: a) controls *versus* cases, and b) patients ≤5 years *versus* those >5* *years of age. ECnNO LAMA: electrochemical nasal nitric oxide measurement during laryngeal mask ventilation.

Figure 2 presents the measurements stratified by age (≤5 *versus* >5 years) and PCD status, showing the overall good consistency of repeated measurements.

The ICCs and CVs are shown in table 3. The ICCs were >0.95 in the total cohort and in both age groups. As the two confidence intervals substantially overlapped, we were unable to determine a statistically significant difference in the ICC between the two age groups. Likewise, we found no significant difference in CVs between these groups (both p>0.5).

**TABLE 3:** Intraclass correlation coefficients (ICCs) and coefficient of variation (CVs) for electrochemical nasal nitric oxide measurement during laryngeal mask ventilation results in the total cohort and by age group (≤5 and >5 years)

	ICC (95% CI)	CV (95% CI)
**All patients (n=52)**	0.974 (0.972–0.990)	0.116 (0.061–0.167)
**Age ≤5 years (n=31)**	0.954 (0.943–0.984)	0.127 (0.051–0.203)
**Age >5 years (n=21)**	0.977 (0.966–0.993)	0.100 (0.042–0.157)

### Diagnostic performance of ECnNO LAMA measurements

**FIGURE 3:**
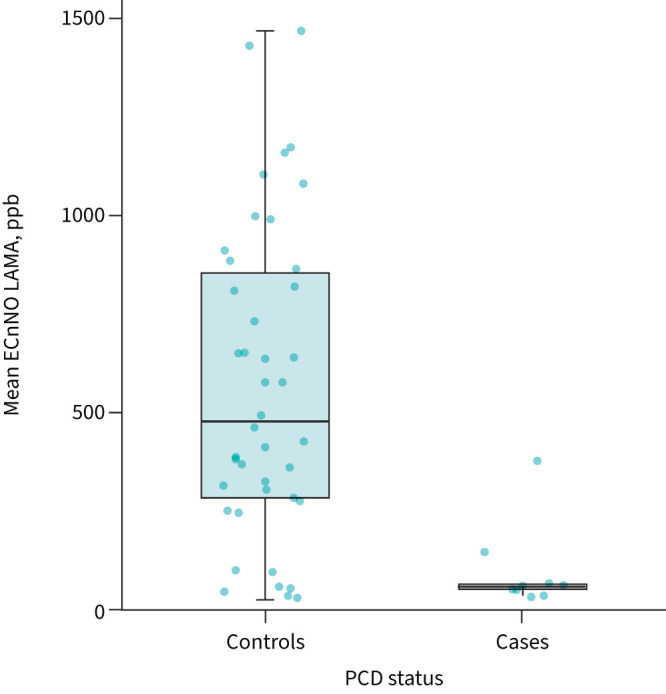
Electrochemical nasal nitric oxide measurement during laryngeal mask ventilation (ECnNO LAMA) in primary ciliary dyskinesia (PCD) *versus* controls. Horizonal bars represent medians, boxes represent interquartile ranges and whiskers represent ranges.

The nNO measurements in our cohort differed significantly between patients with PCD and controls (74.3±38 *versus* 555±396 ppb, p<0.001) (figure 3). The ROC analysis resulted in an area under the curve of 0.89. The optimal cut-off of 178 ppb (flow rate 53.4 nL·min^−1^) yielded a specificity of 0.833 and a sensitivity of 0.900 (figure S3).

## Discussion

To our knowledge, the results of this pilot study represent the first complete dataset on the reproducibility of ECnNO measurement for PCD screening in a paediatric cohort, which includes a substantial proportion of children under the age of 5 years. ECnNO measurements during general anaesthesia with ventilation *via* a laryngeal mask were possible in children of all age groups without complications. Repeated measurements showed considerable agreement and the diagnostic performance indicated the usefulness of the method in screening for PCD.

The repeatability (agreement and precision) of nNO measurements were determined using ICCs and CVs. ICCs were >0.95 in the overall cohort, with no statistical difference between age groups (>5 and ≤5 years), indicating a high level of agreement [17]. Compared with previously reported ICCs for chemiluminescence-based nNO measurements in children aged 5–18 years, the agreement of nNO measurements in our study was higher and, most importantly, independent of the breathing technique used [21].

In terms of precision, the CV in our overall cohort was 11.6% with no significant difference for ages ≥5 or <5 years. Comparison with existing data is challenging, as data on the repeatability of ECnNO measurements are not available for currently available devices. However, there are studies on an ECnNO analyser (NIOX MINO) that is no longer on the market. Using this device, a CV of 15% was documented in a cohort of children and adults (age distribution not specified) using the tidal breathing technique (5 mL·s^−1^, 45 s sampling time with mouth open), with valid measurements in 48 out of 50 subjects [22]. In another cohort of 57 subjects, including two subjects who were <5 years old, the CV for ECnNO was 10% for the same recording technique (tidal breathing, 5 mL·s^−1^; data extracted from results tables) [23]. Thus, our method shows comparable or better precision in a much younger cohort. It is not possible to differentiate whether this is due to a different analysis technique (less flow and less acquisition time required) or to a smaller variation in breath-holding time due to laryngeal breathing *versus* tidal breathing with an open mouth.

Numerous precision data have been published for nNO measured by chemiluminescence, which allow a comparison with our results. As ECnNO LAMA is most comparable to the breath-holding technique, we have focused on this method for comparisons. The reported CV varied from 16.6% in a small cohort of 20 children [24] to 6.7% in a cohort of 282 individuals, 58.5% of whom were <16 years old [25].

In the latter study, outliers (*i.e.* deviating values that were not further defined) “were discarded at the technician's discretion”. The stated CV could therefore underestimate the actual variance that would have resulted if all measurements had been included. We consider it a strength of our study that we have included all measurements without neglecting outliers.

Determining nNO values using breath-holding manoeuvres is extremely challenging in younger children. In a study by Beydon *et al*. [9], technically reliable chemiluminescence measurements during breath-hold, slow expiration against resistance and tidal breathing were possible in as few as 5.6%, 21% and 77% of 105 children under the age of 5 years. CVs in tidal breathing measurements tend to be higher than in velum closure manoeuvres [26, 27].

In summary, the ECnNO LAMA method has a CV comparable to the chemiluminescence technique using velum closure manoeuvres (expiration against resistance and breath-holding) in children aged <5 years. We are unable to determine whether this is due to the electrochemical device itself or due to the measurement setting.

Although this aspect was not the primary focus of the study, ECnNO values were lower in younger children, and there was a large overlap in ECnNO results, especially in the younger patients (figures 1, 2b and S2). This is not surprising because it is well known that nNO values are physiologically lower in the first year of life and during URIs [23]. For this reason, nNO screening values should be confirmed by repeated measurements at this age, especially if pathologically low values are detected.

As with all nNO measurement techniques, the ECnNO technique must be postponed or reassessed if there is evidence of a URI, as was the case in a substantial proportion of all records available in our study (18 out of 70). When describing the characteristics of the measurement, it is important to note that low absolute values in children may be due to young age or clinically silent URI and do not necessarily reflect pathology. Still, repeatability within a series of measurements is not affected.

It has already been noted that nNO screening is limited and that the currently used cut-off values may need to be revised [28]. In our pilot study with multiple measurements in a mixed cohort comprising 10 PCD cases, ECnNO LAMA screening showed a decent ability to find cases, with a specificity of 0.833 and a sensitivity of 0.90. The optimal cut-off value of 178 ppb was found in an ROC analysis. These results should be considered as a proof of concept in our data. Undoubtedly, this needs to be verified by larger studies with complete diagnostics including functional analysis (HSVA) in all patients. We do not recommend using these rather preliminary cut-offs in clinical practice until further validated data are available.

At our regional centre, bronchoscopies are performed as part of our diagnostic workup in selected children with chronic wet cough to rule out anatomical variations and collect lower airway samples, especially in young children who cannot produce sputum. It must be acknowledged, however, that this approach may be unsuitable for other centres and their patient populations. Of course, bronchoscopy is not a mandatory procedure for diagnosing PCD. Nasal brush biopsy can be performed without general anaesthesia even in very young children.

Limitations of our study include its retrospective nature, the relatively small number of cases including three patients with only probable PCD and the lack of HSVA performed at our site. As functional evaluation of ciliary function was only performed externally in a few patients with very high clinical suspicion, other PCD cases could have been missed. This may influence the results of the sensitivity/specificity analysis.

Moreover, although our results show a wide range for possible ECnNO LAMA data, this does not technically exclude effects of the anaesthetics used on the whole cohort, as it is possible that systemic administration of such drugs could affect nitric oxide production in humans in general. However, this should not affect the ECnNO LAMA technique or its characteristics *per se*.

It is essential that the ECnNO LAMA measurements are verified in future studies with larger cohorts and that age-specific cut-off values are determined. A comparison with chemiluminescence-based nNO measurements as the gold standard is required in such studies to determine diagnostic accuracy.

In conclusion, our novel ECnNO LAMA technique is feasible and showed promising repeatability and precision in screening for PCD in children <5 years of age. While further studies are needed, the method may help in the diagnosis of PCD in young patients and simplify a challenging differential diagnostic process.

## Supplementary material

**Please note:** supplementary material is not edited by the Editorial Office, and is uploaded as it has been supplied by the author. Supplementary material 01018-2024.SUPPLEMENT

## References

[1] Leigh MW, Ferkol TW, Davis SD, et al. Clinical features and associated likelihood of primary ciliary dyskinesia in children and adolescents. Ann Am Thorac Soc 2016; 13: 1305–1313. doi:10.1513/AnnalsATS.201511-748OC 27070726 PMC5021075

[2] Shapiro AJ, Davis SD, Polineni D, et al. Diagnosis of primary ciliary dyskinesia. An official American Thoracic Society clinical practice guideline. Am J Respir Crit Care Med 2018; 197: e24–e39. doi:10.1164/rccm.201805-0819ST 29905515 PMC6006411

[3] Lucas JS, Barbato A, Collins SA, et al. European Respiratory Society guidelines for the diagnosis of primary ciliary dyskinesia. Eur Respir J 2017; 49: 1601090. doi:10.1183/13993003.01090-2016 27836958 PMC6054534

[4] Beydon N, Kouis P, Marthin JK, et al. Nasal nitric oxide measurement in children for the diagnosis of primary ciliary dyskinesia: European Respiratory Society technical standard. Eur Respir J 2023; 61: 2202031. doi:10.1183/13993003.02031-2022 36822632

[5] Shapiro AJ, Dell SD, Gaston B, et al. Nasal nitric oxide measurement in primary ciliary dyskinesia. A technical paper on standardized testing protocols. Ann Am Thorac Soc 2020; 17: e1–e12. doi:10.1513/AnnalsATS.201904-347OT 31770003 PMC6993796

[6] Manna A, Montella S, Maniscalco M, et al. Clinical application of nasal nitric oxide measurement in pediatric airway diseases. Pediatr Pulmonol 2015; 50: 85–99. doi:10.1002/ppul.23094 25156952

[7] Circassia. 2023. NIOX VERO Nasal user manual German. Date last accessed: 22 August 2025.

[8] Circassia. 2023. Product labeling summary/package insert NIOX VERO. Date last accessed: 22 August 2025.

[9] Beydon N, Tamalet A, Escudier E, et al. Breath-holding and tidal breathing nasal NO to screen children for primary ciliary dyskinesia. Pediatr Pulmonol 2021; 56: 2242–2249. doi:10.1002/ppul.25432 33860637

[10] Holgersen MG, Marthin JK, Nielsen KG. Proof of concept: very rapid tidal breathing nasal nitric oxide sampling discriminates primary ciliary dyskinesia from healthy subjects. Lung 2019; 197: 209–216. doi:10.1007/s00408-019-00202-x 30762092

[11] Shapiro AJ, Josephson M, Rosenfeld M, et al. Accuracy of nasal nitric oxide measurement as a diagnostic test for primary ciliary dyskinesia. A systematic review and meta-analysis. Ann Am Thorac Soc 2017; 14: 1184–1196. doi:10.1513/AnnalsATS.201701-062SR 28481653 PMC6137897

[12] Harris A, Phan H, Borca F, et al. Experience of measuring nasal nitric oxide (nNO) in a National Primary Ciliary Dyskinesia (PCD) Centre (2006-20). Eur Respir J 2020; 26: Suppl. 64, 2070. doi:10.1183/13993003.congress-2020.2070

[13] Lucas J, Leigh M, Beydon N, et al. New portable nasal nitric oxide (nNO) analyser differentiates primary ciliary dyskinesia (PCD) from healthy individuals. Eur Respir J 2017; 50: Suppl. 61, PA1848. doi:10.1183/1393003.congress-2017.PA1848

[14] Rickard K. 2017. NIOX VERO nasal application in primary ciliary dyskinesia: NCT02622061. Date last accessed: 22 August 2025. Date last updated: 15 May 2017.

[15] Werner C, Lablans M, Ataian M, et al. An international registry for primary ciliary dyskinesia. Eur Respir J 2016; 47: 849–859. doi:10.1183/13993003.00776-2015 26659107

[16] Lin L. Overview of agreement statistics for medical devices. J Biopharm Stat 2008; 18: 126–144. doi:10.1080/10543400701668290 18161545

[17] McBride GB. A proposal for strength-of-agreement criteria for Lin's concordance correlation coefficient. NIWA Client Report HAM2005-062, 2005. Date last accessed: 22 August 2025. www.medcalc.org/download/pdf/McBride2005.pdf.

[18] Robin X, Turck N, Hainard A, et al. pROC: an open-source package for R and S+ to analyze and compare ROC curves. BMC Bioinformatics 2011; 12: 77. doi:10.1186/1471-2105-12-77 21414208 PMC3068975

[19] Carrasco JL, Martinez JP. cccrm: concordance correlation coefficient for repeated (and non-repeated) measures. Date last accessed: 22 August 2025. Date last updated: 22 July 2025.

[20] Wickham H. ggplot2: Elegant Graphics for Data Analysis. New York, Springer-Verlag, 2016.

[21] Mateos-Corral D, Coombs R, Grasemann H, et al. Diagnostic value of nasal nitric oxide measured with non-velum closure techniques for children with primary ciliary dyskinesia. J Pediatr 2011; 159: 420–424. doi:10.1016/j.jpeds.2011.03.007 21514598

[22] Harris A, Bhullar E, Gove K, et al. Validation of a portable nitric oxide analyzer for screening in primary ciliary dyskinesias. BMC Pulm Med 2014; 14: 18. doi:10.1186/1471-2466-14-18 24507708 PMC3929562

[23] Marthin JK, Philipsen MC, Rosthoj S, et al. Infant nasal nitric oxide over time: natural evolution and impact of respiratory tract infection. Eur Respir J 2018; 51: 1702503. doi:10.1183/13993003.02503-2017 29748307

[24] Kharitonov SA, Walker L, Barnes PJ. Repeatability of standardised nasal nitric oxide measurements in healthy and asthmatic adults and children. Respir Med 2005; 99: 1105–1114. doi:10.1016/j.rmed.2005.02.019 16085212

[25] Marthin JK, Nielsen KG. Choice of nasal nitric oxide technique as first-line test for primary ciliary dyskinesia. Eur Respir J 2011; 37: 559–565. doi:10.1183/09031936.00032610 20525709

[26] Beydon N. Nasal nitric oxide measurement variability to establish a standard for reliable results. ERJ Open Res 2022; 8: 00028-2022. doi:10.1183/23120541.00028-2022 35769413 PMC9234436

[27] Beydon N, Chambellan A, Alberti C, et al. Technical and practical issues for tidal breathing measurements of nasal nitric oxide in children. Pediatr Pulmonol 2015; 50: 1374–1382. doi:10.1002/ppul.23167 25731630

[28] Raidt J, Krenz H, Tebbe J, et al. Limitations of nasal nitric oxide measurement for diagnosis of primary ciliary dyskinesia with normal ultrastructure. Ann Am Thorac Soc 2022; 19: 1275–1284. doi:10.1513/AnnalsATS.202106-728OC 35202559

