# Supplementary material for: Electrochemical nasal nitric oxide measurement during laryngeal mask ventilation as primary ciliary dyskinesia screening
Source: ERJ Open Res. 2025 Nov 10;11(6):01018-2024. doi: 10.1183/23120541.01018-2024 (PMC12598594; doi:10.1183/23120541.01018-2024)
Supplement: Supplementary file 1 [file 01018-2024.SUPPLEMENT.pdf]

Supplementary Data for manuscript:

Electrochemical nasal NO measurement during laryngeal mask ventilation as PCD screening

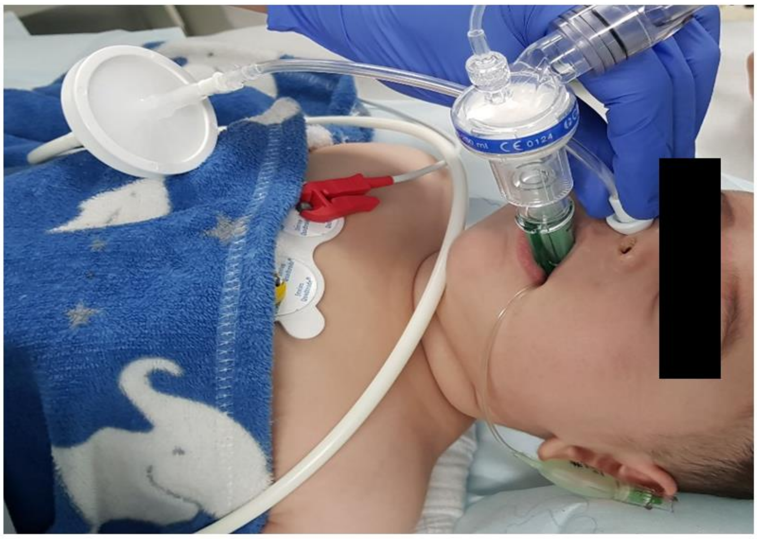

Figure S1, supplementary material: setting of ECnNO LAMA measurement. Patient on ventilation via laryngeal mask. Olive of device attached to patient's nose

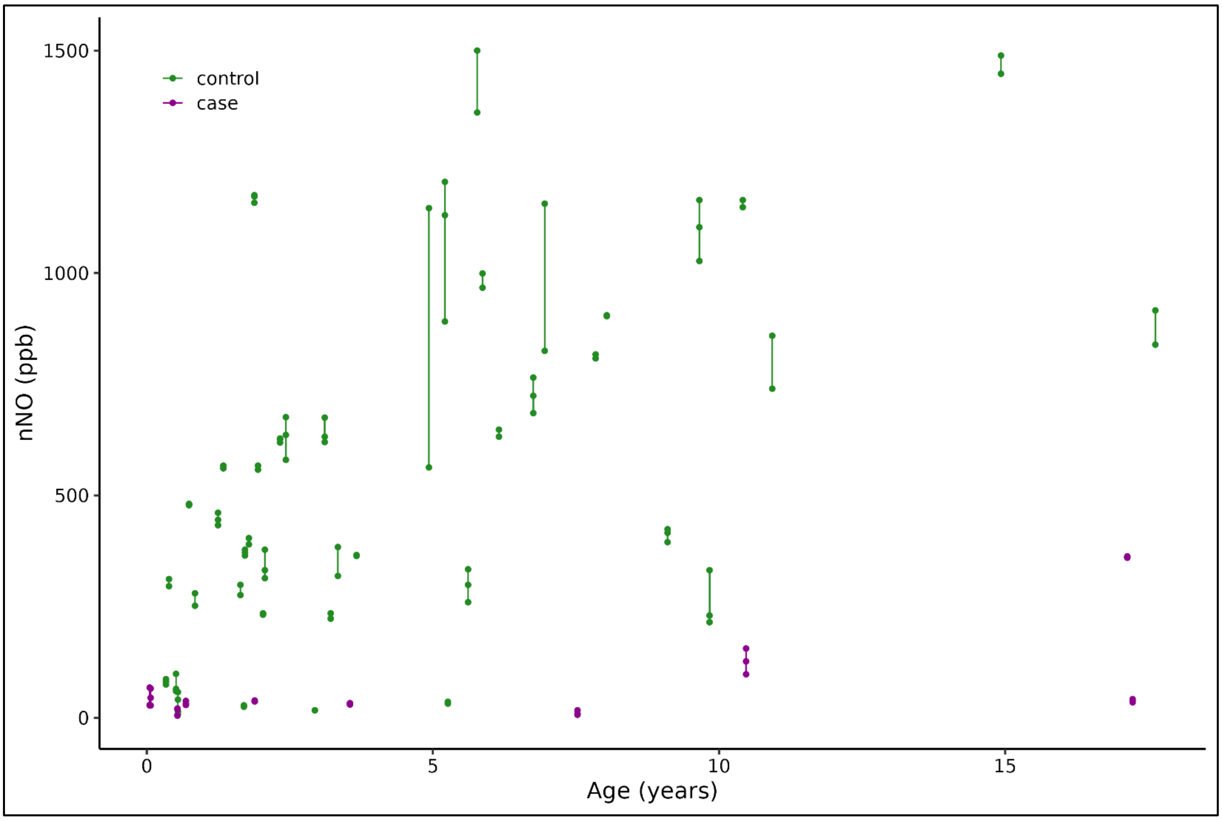

Figure S2, supplementary material: nNO vs. age per controls and cases . Note: here, all patients are included. Compare Figure 2 in main text.

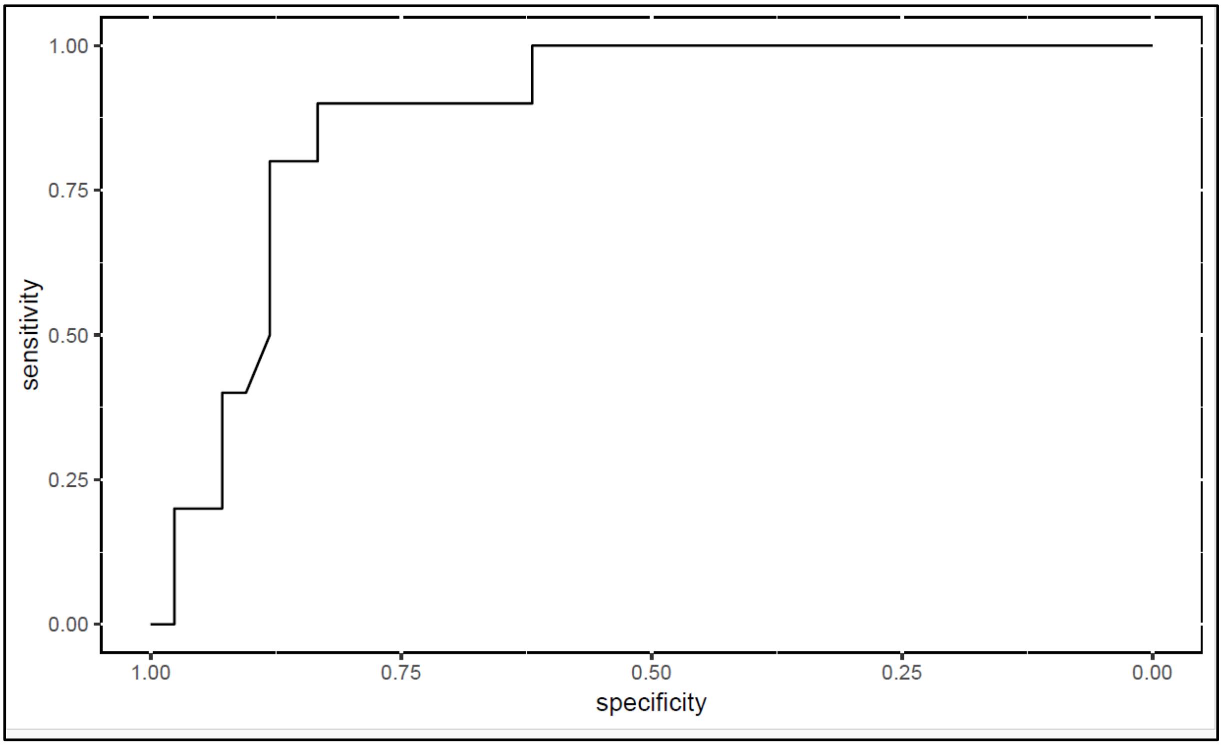

Figure S3, Supplementary material: ROC analysis for ECnNO

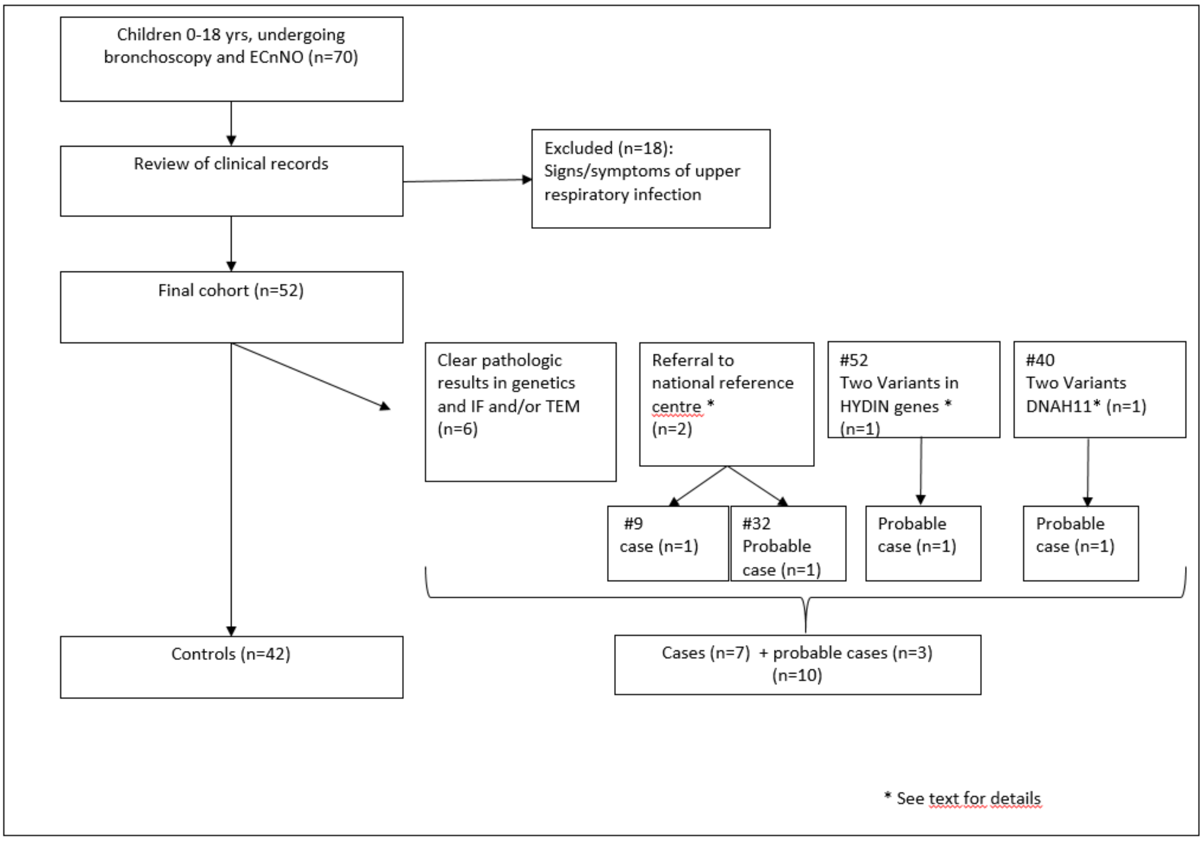

Figure S4, Supplementary material: Flow chart for study population.

[For change log: added numbers of patients without clear pathologic findings, added "cases +" in last row]

Supplementary data to table 2 and figure S4:

I. List of genes tested for in genetic analysis:

DNAH5, DNAH9, DNAH11, DNAII ,DNA12, NME8, DNALI, CCDC103, CCDC114, ARMC4, CCDCl51 , TTC25, DNAAF1, DNAAF2, DNAAF3,DNAAF4, DNAAF5, DNAAF6, DNAAF7, CFAP298, SPAG1, LRRC6, CFAP300, HYDIN, SPEF2, CFAP221 , DNAJBl3, RSPH1, RSPH3, RSPH4A, RSPH9, SPEF2, STK36, DRC1, DRC2, DRC4, CCDC39, CCDC40, RPGR, OFD1, CFAP74, GAS2L2, MNSI, CCNO, MCIDAS und FOXJ1.

II. Discussion of Patients

(1) #9: this patient was referred in person to the national reference centre. We cite from the report: "The first variant is a classic loss-of-function mutation. The second variant is an amino acid exchange. It is possible that the second variant also influences splicing. The segregation analysis of the parental DNA has now demonstrated the compound heterozygous inheritance. (…) The second variant is most likely a pathogenic mutation. The cilia beat analysis also revealed a conspicuous finding: the nasal NO production rate was slightly reduced in the offline measurement. Overall, further diagnostics confirmed the strong suspicion of primary ciliary dyskinesia (…) Since the latter DNA variant (the amino acid exchange) may not lead to a complete loss of gene

function, an abnormal immunofluorescence microscopic finding is not necessarily to be expected “.

(2) #32: Patient no. 32, too, was personally examined directly by the national reference centre. Citing from the original report, the colleagues stated: “with the results [comment by authors: as seen in Table 2] we cannot confirm PCD diagnosis per 100%., but we still consider the diagnosis of PCD to be probable.”

(3) #40 – discussion of genetic variants:

variant 1: c.5778+1G>A, p?

- frequency in general population 0,002% (4x in GnomAD)
- ClinVar 454688, herein reported as pathogen once
- the variant is truncating
- The variant has already been detected in a non-related affected person and reported as pathogenic (PS4_SUP, ClinVar)
- The variant is associated with a highly specific and matching phenotype (PP4_SUP)

variant 2: c.9765A>T, p.(Leu3255Phe)

- in silico: uniformly pathogenic (PP3), weakly conserved amino acid
- The variant is associated with a highly specific and matching phenotype (PP4_SUP)
- At the affected amino acid position, another amino acid exchange is already described as pathogenic in the databases for disease-causing variants and in the literature (PM5_MOD, HGMD, CM1110362, Knowles et al, 2012, PMID: 22184204)
- Although there are indications of clinical relevance, the variant must still be assessed as being of unclear significance.

(4) #52:

Genetic testing revealed two variants, both of which were classified as variants of unknown significance (VUS). It is unknown whether the variants affect protein function. Methodologically, it was impossible to clarify in which of the four known HYDIN-alleles the variants are located.

The authors decided to classify this patient as “probable case” based on the very suggestive clinical impression and the assessment of the variants in silico and via a rare allele frequency in the general population (gnomAD v4[1]). HYDIN mutant respiratory cilia lack the C2b projection of the central pair (CP) apparatus*[2]*. Variant 1 is listed once in ClinVar as a VUS.

[1] https://gnomad.broadinstitute.org/

[2] Olbrich H, Schmidts M, Werner C, Onoufriadis A, Loges NT, Raidt J, Banki NF, Shoemark A, Burgoyne T, Al Turki S, Hurles ME; UK10K Consortium; Köhler G, Schroeder J, Nürnberg G, Nürnberg P, Chung EM, Reinhardt R, Marthin JK, Nielsen KG, Mitchison HM, Omran H. Recessive HYDIN mutations cause primary ciliary dyskinesia without randomization of left-right body asymmetry. Am J Hum Genet. 2012 Oct 5;91(4):672-84. doi

It should be noted, however, that due to the paralogous segments (HYDIN 1 and 2) [2] it is unclear whether a change results at the protein level especially in the case of a compound heterozygous constellation on the background of possibly three wild-type alleles.

a. Variant 1:
   - The allele frequency is stated to be 0.002566
   - In silico prediction: A majority of prediction tools support a pathogenic prediction including CADD. Revel shows an "indeterminate" result.

b. Variant 2:
   - The variant is not found in gnomAD, indicating a very rare variant.
   - In silico prediction: indeterminate across all available resources

Additionally, please see Fig S5 for details.

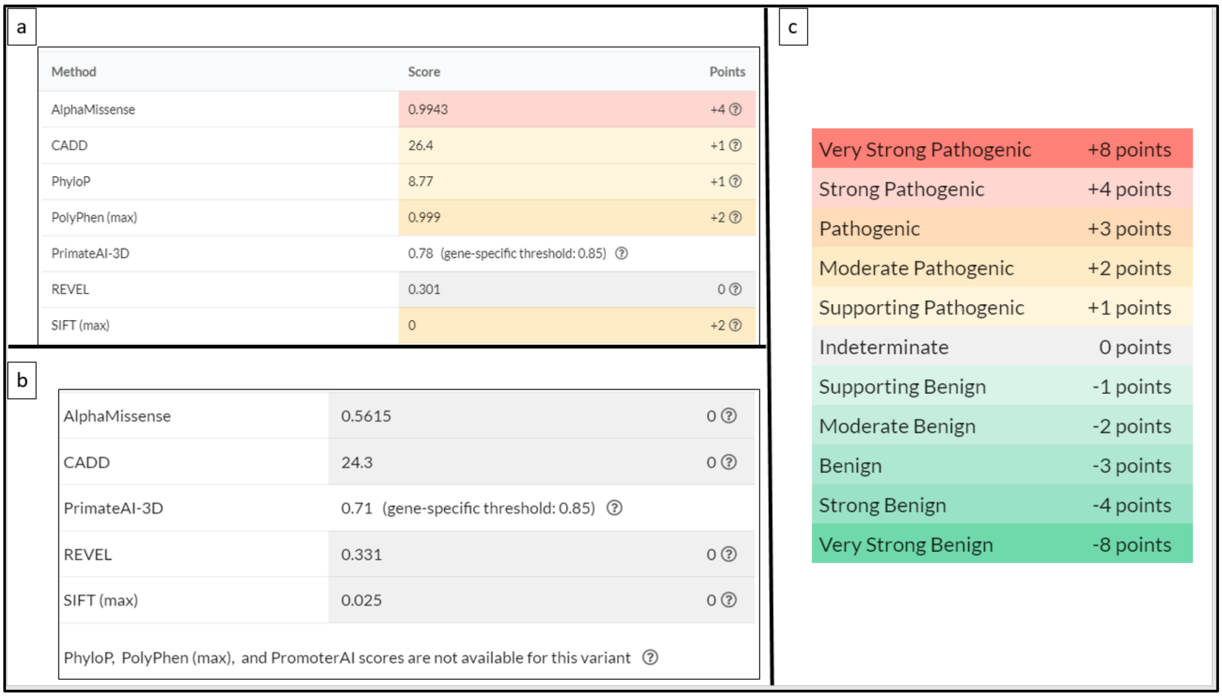

a

Method	Score	Points
AlphaMissense	0.9943	+4 ⓘ
CADD	26.4	+1 ⓘ
PhyloP	8.77	+1 ⓘ
PolyPhen (max)	0.999	+2 ⓘ
PrimateAI-3D	0.78 (gene-specific threshold: 0.85) ⓘ	
REVEL	0.301	0 ⓘ
SIFT (max)	0	+2 ⓘ

b

AlphaMissense	0.5615	0 ⓘ
CADD	24.3	0 ⓘ
PrimateAI-3D	0.71 (gene-specific threshold: 0.85) ⓘ	
REVEL	0.331	0 ⓘ
SIFT (max)	0.025	0 ⓘ
PhyloP, PolyPhen (max), and PromoterAI scores are not available for this variant ⓘ		

c

Very Strong Pathogenic	+8 points
Strong Pathogenic	+4 points
Pathogenic	+3 points
Moderate Pathogenic	+2 points
Supporting Pathogenic	+1 points
Indeterminate	0 points
Supporting Benign	-1 points
Moderate Benign	-2 points
Benign	-3 points
Strong Benign	-4 points
Very Strong Benign	-8 points

Fig S5 a/b/c: in silico predicition results for genetic variants of patient #52. Panel a) for Variant 1, panel b) for variant 2, legend in panel c) based on thresholds and points established in Bergquist et al. 2024 [1] and Pejaver et al. 2022[2].

1 Timothy Bergquist, Sarah L. Stenton, Emily A.W. Nadeau, Alicia B. Byrne, Marc S. Greenblatt, Steven M. Harrison, Sean V. Tavtigian, Anne O'Donnell-Luria, Leslie G. Biesecker, Predrag Radivojac, Steven E. Brenner, Vikas Pejaver, ClinGen Sequence Variant Interpretation Working Group Calibration of additional computational tools expands ClinGen recommendation options for variant classification with PP3/BP4 criteria. bioRxiv 2024.09.17.611902; doi: https://doi.org/10.1101/2024.09.17.611902

2 Pejaver V, Byrne AB, Feng BJ, Pagel KA, Mooney SD, Karchin R, O'Donnell-Luria A, Harrison SM, Tavtigian SV, Greenblatt MS, Biesecker LG, Radivojac P, Brenner SE; ClinGen Sequence Variant Interpretation Working Group. Calibration of computational tools for missense variant pathogenicity classification and ClinGen recommendations for PP3/BP4 criteria. Am J Hum Genet. 2022 Dec 1;109(12):2163-2177. doi: 10.1016/j.ajhg.2022.10.013. Epub 2022 Nov 21. PMID: 36413997; PMCID: PMC9748256.
